# Dialysis timing may be deferred toward very late initiation: An observational study

**DOI:** 10.1371/journal.pone.0233124

**Published:** 2020-05-13

**Authors:** Yun-Lun Chang, Jie-Sian Wang, Hung-Chieh Yeh, I-Wen Ting, Han-Chun Huang, Hsiu-Yin Chiang, Chiung-Tzu Hsiao, Pei-Lun Chu, Chin-Chi Kuo

**Affiliations:** 1 Division of Nephrology, Department of Internal Medicine, China Medical University Hospital and College of Medicine, China Medical University, Taichung, Taiwan; 2 Big Data Center, China Medical University Hospital and College of Medicine, China Medical University, Taichung, Taiwan; 3 Department of Laboratory Medicine, China Medical University Hospital, Taichung, Taiwan; 4 Department of Medical Laboratory Science and Biotechnology, China Medical University, Taichung, Taiwan; 5 Division of Nephrology, Department of Internal Medicine, Fu Jen Catholic University Hospital, New Taipei City, Taiwan; 6 School of Medicine, Fu Jen Catholic University, New Taipei City, Taiwan; University of Catanzaro, ITALY

## Abstract

The optimal timing to initiate dialysis among patients with an estimated glomerular filtration rate (eGFR) of <5 mL/min/1.73 m^2^ is unknown. We hypothesized that dialysis initiation time can be deferred in this population even with high uremic burden. A case-crossover study with case (0–30 days before dialysis initiation [DI]) and control (90–120 days before DI) periods was conducted in 1,079 hemodialysis patients aged 18–90 years at China Medical University Hospital between 2006 and 2015. The uremic burden was quantified based on 7 uremic indicators that reached the predefined threshold in case period, namely hemoglobin, serum albumin, blood urea nitrogen, serum creatinine, potassium, phosphorus, and bicarbonate. Dialysis timing was classified as standard (met 0–2 uremic indicators), late (3–5 indicators), and very late (6–7 indicators). Median eGFR-DI of the 1,079 patients was 3.4 mL/min/1.73 m^2^ and was 2.7 mL/min/1.73 m^2^ in patients with very late initiation. The median follow-up duration was 2.42 years. Antibiotics, diuretics, antihypertensive medications, and non-steroidal anti-inflammatory drugs (NSAIDs) were more prevalently used during the case period. The fully adjusted hazards ratios of all-cause mortality for the late and very late groups were 0.97 (95% confidence interval 0.76–1.24) and 0.83 (0.61–1.15) compared with the standard group. It is safe to defer dialysis initiation among patients with chronic kidney disease (CKD) having an eGFR of <5 mL/min/1.73 m^2^ even when patients having multiple biochemical uremic burdens. Coordinated efforts in acute infection prevention, optimal fluid management, and prevention of accidental exposure to NSAIDs are crucial to prolong the dialysis-free survival.

## Introduction

Clinical judgement to initiate dialysis in patients with stage-5 chronic kidney disease (CKD) remains an “art of medicine” decision. The only randomized trial, the Initiating Dialysis Early and Late (IDEAL) study [1], and the following observation studies including 1 meta-analysis have suggested no survival benefit of initiating dialysis early, which was defined as a range of the estimated glomerular filtration rate (eGFR) at dialysis initiation (eGFR-DI) of >10 mL/min/1.73 m^2^ [2–7]. In light of emerging evidence, the international practice pattern of dialysis initiation has moved from eGFR-DI of >10 mL/min/1.73 m^2^ in the 2000s toward close to 7 mL/min/1.73 m^2^ in the 2010s [8–11]. Furthermore, the recent practice guidelines in Nephrology endorse the “safe intent-to-defer” approach rather than the specific eGFR threshold-based approach [12–15]. However, to what extent dialysis initiation can be safely deferred lacks robust evidence-based data. For instance, only 1 study has determined the outcome of eGFR-DI of <5 mL/min/1.73 m^2^ or even lower [16].

Moving in the direction of personalized dialysis initiation in patients with stage-5 CKD makes it impractical to differentiate early and late initiation based on an eGFR-DI threshold. In real practice, the main concerns of nephrologists are not only biochemical abnormalities, such electrolyte abnormalities or elevated serum creatinine (S-Cre), but also uremic symptoms, particularly dyspnea from fluid overload, refractory nausea/vomiting, or sleep disturbances [17]. The trigger of commencement of dialysis is usually when objective biochemical data correlate well with the subjectively reported symptoms that are refractory to medical control. In case of any discrepancy between biochemical numbers and clinical symptoms, dialysis planning will be adjusted between proactive and reactive risk control. For example, for patients with severe heart failure, proactive dialysis may be initiated for better management of cardiopulmonary distress rather than uremic symptoms. By contrast, dialysis may be safely deferred in symptom-free patients even with an eGFR consistently below 5 mL/min/1.73 m^2^.

Taiwan, the country with the highest prevalence and incidence of end-stage renal disease (ESRD) in the world, has developed a strict definition of a catastrophic ESRD status and a corresponding hemodialysis practice guideline, setting an absolute eGFR threshold of <5 mL/min/1.73 m^2^ since 2000. According to a study in Taiwan, the median eGFR-DI in national registry data, which was 4.7mL/min/1.73 m^2^, well reflected this country’s current practice and timing is much “later” than the late group defined by the IDEAL trial [1, 16]. The finding of overall low mortality is in agreement with the recent consensus in dialysis timing—earlier is not better. In the present study, we applied a case-time-control study to minimize lead-time bias in a hemodialysis population of a tertiary medical center and used the dynamic changes in uremic indicators such as serum phosphorus, albumin, and bicarbonate, in addition to eGFR, to quantify uremic burden. We hypothesized that dialysis timing can be deferred even among patients with stage-5 CKD with a high uremic burden.

## Methods

### Study population

In 2017, the Big Data Center and the Office of Information Technology of China Medical University Hospital (CMUH) established the CMUH Clinical Research Data Repository (CRDR), which carefully verifies and validates data from various clinical sources to unify trackable patient information generated during the healthcare process. Between January 1, 2003 and December 31, 2016, the CMUH-CRDR accumulated the single unified views of 2,660,472 patients who had sought care at CMUH. Patient information includes data on administration and demography, diagnosis, medical and surgical procedures, prescriptions, laboratory measurements, physiological monitoring data, hospitalization, and catastrophic illness status [18]. The interoperability of the CMUH-CRDR has further expanded access to national population-based health-related databases (e.g., mortality database), which are systematically maintained by the Health and Welfare Data Science Center of the Ministry of Health and Welfare. All patients enrolled in the CMUH-CRDR were followed up until December 31, 2016 or death, whichever occurred first. The present study cohort comprised 1,079 hemodialysis patients aged 18–90 years with continual care at CMUH hemodialysis center between 2006 and 2015. **S1 Fig** provides an overview of patient selection as well as the exclusion criteria. The Research Ethical Committee/Institutional Review Board of China Medical University Hospital approved this study and waived the requirement for informed consent because this was a retrospective secondary data analysis (CMUH105-REC3-068).

### Case-crossover design

**Fig 1:**
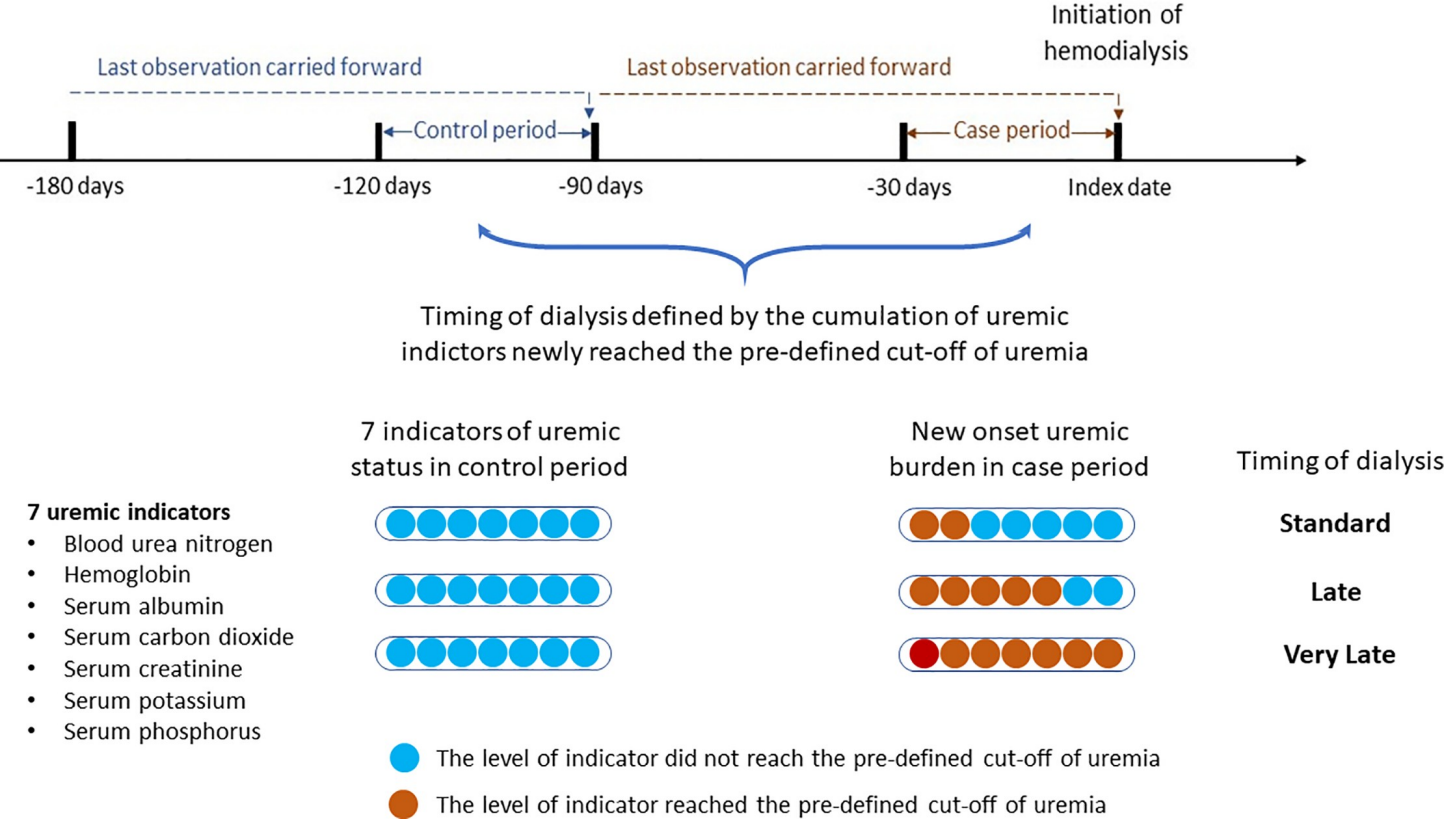
Case-crossover study design and timing of exposure measurements including uremic indicators and medications in relation to hemodialysis initiation. Dialysis timing was classified as standard (0–2 uremic indicators newly reached the predefined cutoffs during the case period, No-to-Yes group), late (3–5 indicators), and very late (6–7 indicators).

In the present study, we used a case-crossover design to quantify uremic burden before dialysis initiation. This design has the advantage of controlling unmeasured time-invariant confounders, such as environmental exposures, drug adherence, and dietary factors [19]. Each patient acted as his or her own control. The case period of each patient was defined as 0–30 days before dialysis initiation, and the matched control period was defined as 90–120 days before dialysis initiation (**Fig 1**). Uremic burden was quantified by 7 dialysis indicators in the case period, namely hemoglobin, serum albumin, blood urea nitrogen (BUN), S-Cre, potassium, phosphorus, and bicarbonate. If data were unavailable within the case or control period, the last available laboratory values within 60 days before the case or control period were used. The cutoff values of dialysis indicators were <9.0 g/dL for hemoglobin, <3.5 g/dL for albumin, >100 mg/dL for BUN, >10 mg/dL for S-Cre, >5.5 mmol/L for potassium, >6.5 mg/dL for phosphorus, and <20 mmol/L for carbon dioxide (CO_2_) [20]. Dialysis timing was classified as standard (0–2 uremic indicators newly reached the predefined cutoffs during the case period, No-to-Yes group), late (3–5 indicators), and very late (6–7 indicators) (**S1 Table and Fig 1**).

### Laboratory measurements and covariables

CMUH’s clinical laboratory has received full accreditation from the Taiwan Accreditation Foundation and the College of American Pathologists Laboratory Accreditation Program since September 2003 and December 2008, respectively. Hemoglobin concentration was measured using an automatic analyzer Sysmex HST-302N (Sysmex HST-series, Kanogawa, Japan). The reference ranges for hemoglobin in men and women are 13.7–17.0 g/dL and 11.1–15.0 g/dL, respectively. Serum phosphorus levels were measured using the timed endpoint colorimetric method, and creatinine levels were measured using the Jaffe rate method (kinetic alkaline picrate) with a Beckman UniCel® DxC 800 (Beckman Coulter Inc., CA, USA) at CMUH Central Laboratory. We used serum CO_2_ to represent acid–base status, which was determined by the Henderson–Hasselbalch equation. For patients whose acid–base status was evaluated through blood gas bicarbonate, we converted venous and arterial bicarbonate to total CO_2_ by adding 2 and 3 mmol/L, respectively [21]. eGFR was estimated using the abbreviated Chronic Kidney Disease Epidemiology Collaboration (CKD-EPI) equation [22]. Kt/V was calculated using the Daugirdas equation [23]. Vascular accesses used on first dialysis were categorized into fistulas, grafts, and dialysis catheters. Registry data or information obtained from electronic medical records (EMRs) within a 1-year window before enrollment was used to compile baseline comorbidities, relevant biochemical measures, and medication use including lipid-lowering, glucose-lowering, anti-hypertensive, and potential nephrotoxic agents such as nonsteroidal anti-inflammatory drugs (NSAIDs), contrast, beta-lactam antimicrobials, sulfonamides, and vancomycin. Indications of diabetes mellitus and hypertension were based on the clinical diagnosis of physicians using the International Classification of Disease, Revision 9, Clinical Modification diagnosis code and the use of glucose-lowering/anti-hypertensive agents. A history of cardiovascular disease (CVD) was defined as coronary artery disease, myocardial infarction, stroke, or heart failure documented in EMRs. The definitions for some of the covariates were used in our previous work [18, 24–26].

### Statistical analysis

Continuous variables were expressed as medians and interquartile ranges and compared using the nonparametric Kruskal–Wallis test, whereas categorical variables were expressed as frequency (percentage) and compared using the chi-square test. Four transition patterns between case and control periods were noted for each dialysis indicator and medication: yes-yes, yes-no, no-yes, and no-no. The 1-year trajectories of the 7 dialysis indicators before dialysis were modeled using group-based trajectory modeling [27–29]. To evaluate the association between medication exposure and dialysis initiation, the Mantel–Haenszel odds ratio for matched pairs was used as the measure of analysis [19]. Due to the large amount of missing data of serum albumin up to 11.4%, we further performed multiple imputation with fully conditional method (FCS) method in R, an iterative Markov chain Monte Carlo (MCMC) procedure, to replace the missing values for albumin, hemoglobin, and BMI with imputed values. We specified the number of imputation as 20 and the number of iteration as 100. We then specified estimation model on each of the 20 imputed databases followed by combining these estimates to obtain one set of inferential statistics. The findings based on the imputed database were consistent with the results of the original “available case analysis”. The associations between each group of dialysis initiation and all-cause mortality were estimated through multivariable Cox regression analysis. Selection of potential confounding factors for adjustment was based on a priori knowledge. All statistical analyses were performed in SAS version 9.4 (SAS Institute Inc., Cary, NC, USA) and R version 3.6.1 (R Foundation for Statistical Computing, Vienna, Austria). The 2-sided statistical significance level was set at α = 0.05.

## Results

**Fig 2:**
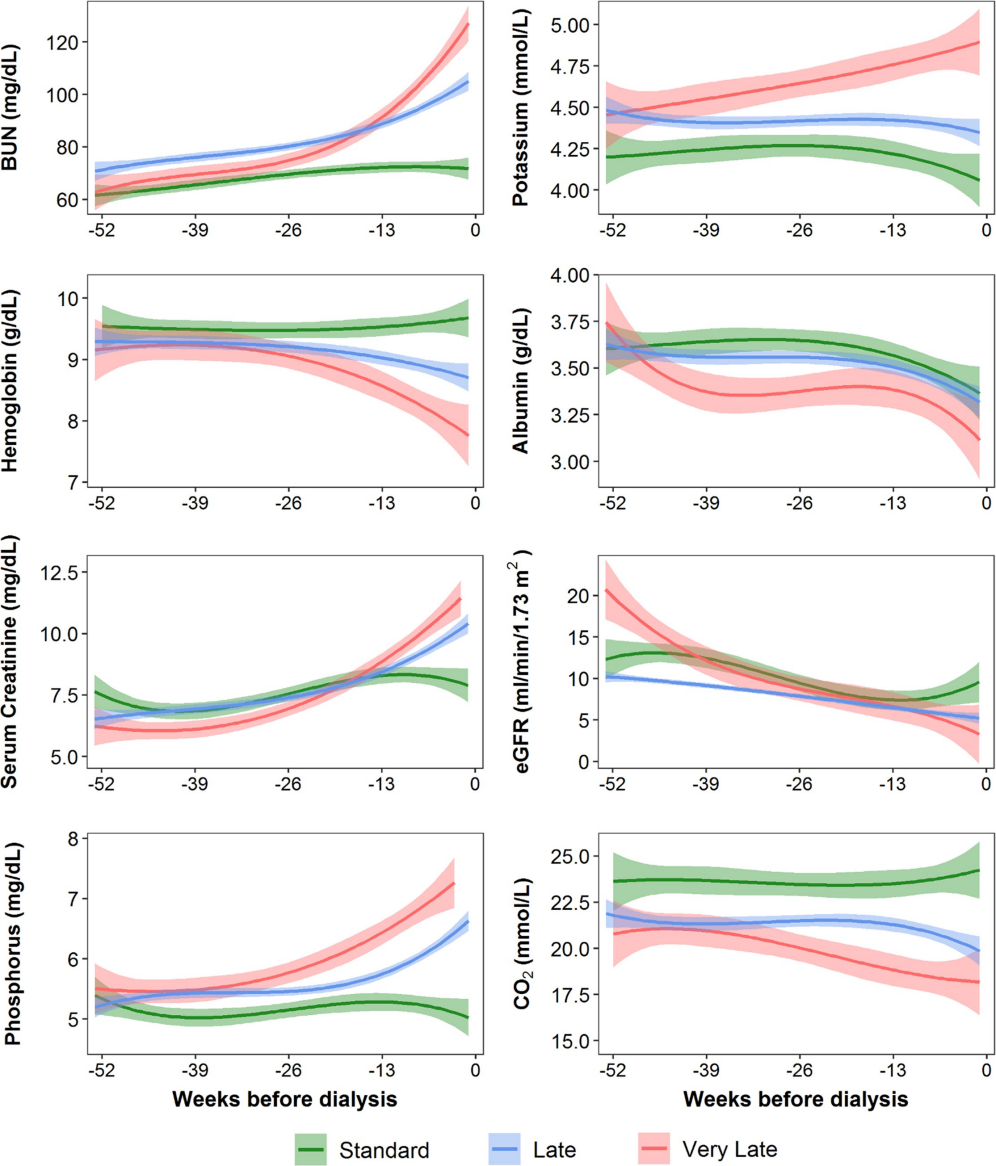
Longitudinal trajectories of the 7 common uremic indicators 1 year before hemodialysis initiation based on the 3 groups of dialysis timing (standard [green], late [blue], and very late [red]).

The transition patterns of crucial indicators of initiating chronic dialysis of 1,079 patients with ESRD are provided in **S1 Table**. The median follow-up duration was 2.42 years. In contrast to common belief, persistent or newly developed uncontrolled hyperkalemia (serum potassium > 5 mmol/L) is not the main trigger factor (no-to-yes, 19.0%) of hemodialysis initiation. Instead, a new event of S-Cre > 10 mg/dL or BUN > 100 mg/dL is the main consideration for clinicians at our hospital to start hemodialysis, followed by hyperphosphatemia (**S1 Table**). In the control period, the three groups (standard, late, and very late) had comparable S-Cre and eGFR (**Table 1**). Tracking the 1-year trajectories of these dialysis initiation indicators, patients in the very late group consistently had the worst biochemical profiles (**Fig 2**), which well supports the rationality of our classification.

**Table 1:** Baseline demographics and clinical characteristics based on the timing of dialysis initiation.

Variables	Total (*N* = 1079)	Standard (*n* = 196)	Late (*n* = 659)	Very Late (*n* = 224)	*p*-value^a^	*p*-value for trend^b^
Man, n (%)	561 (52.0)	85 (43.4)	348 (52.8)	128 (57.1)	0.015	0.005
Age at entry (year), median (IQR)	61.4 (51.3, 71.2)	63.0 (53.8, 74.0)	61.8 (51.6, 71.7)	57.7 (47.6, 68.5)	0.001	< 0.001
Body mass index (kg/m^2^)	23.5 (21.5, 26.5)	23.6 (21.2, 26.4)	23.5 (21.64, 26.3)	23.5 (21.5, 26.7)	0.591	0.322
Comorbidity, n (%)						
Cardiovascular disease	450 (41.7)	94 (48.0)	280 (42.5)	76 (33.9)	0.012	0.003
Hypertension	867 (80.4)	142 (72.4)	534 (81.0)	191 (85.3)	0.003	0.001
Diabetes	549 (50.9)	93 (47.4)	337 (51.1)	119 (53.1)	0.498	0.251
Biochemical profiles in the control periods, median (IQR)						
Blood urea nitrogen (mg/dL)	84 (66, 101)	72 (55, 90)	86 (69, 103)	88 (71, 109)	<0.001	<0.001
Potassium (mmol/L)	4.6 (4.1, 5.1)	4.5 (3.9, 5.1)	4.5 (4.0, 5.0)	5.0 (4.4, 5.4)	<0.001	<0.001
Hemoglobin (g/dL)	9.0 (7.9, 10.1)	9.9 (8.3, 10.5)	9.0 (7.9, 10.1)	8.4 (7.2, 9.5)	<0.001	<0.001
Albumin (g/dL)	3.5 (3.2, 4.0)	3.6 (3.2, 4.1)	3.6 (3.2, 4.0)	3.4 (3.1, 3.8)	0.028	0.014
Phosphorus (mg/dL)	5.6 (4.8, 6.5)	5.4 (4.5, 6.1)	5.5 (4.8, 6.5)	5.9 (5.3, 7.2)	<0.001	<0.001
Serum creatinine (mg/dL)	8.1 (6.2, 10.0)	8.1 (6.1, 9.8)	8.1 (6.0, 10.2)	8.2 (6.4, 10.0)	0.910	0.687
eGFR (mL/min/1.73m2)	5.5 (4.1, 7.6)	5.3 (4.1, 7.0)	5.6 (4.1, 7.5)	5.8 (4.3, 7.8)	0.914	0.741
CO_2_ (mmol/L)	21.1 (17.8, 24.2)	23.5 (19.4, 26.1)	21.2 (17.9, 23.8)	18.4 (16.2, 21.4)	<0.001	<0.001
Biochemical profiles in the case periods, median (IQR)						
Blood urea nitrogen (mg/dL)	125 (97, 157)	84 (63.2, 98)	126 (102, 156)	155 (130, 182.5)	< 0.001	< 0.001
Potassium (mmol/L)	4.9 (4.3, 5.5)	4.4 (4.0, 5.2)	4.8 (4.3, 5.4)	5.7 (5.2, 6.2)	< 0.001	< 0.001
Hemoglobin (g/dL)	7.9 (6.7, 9.1)	9.4 (8.3, 10.3)	7.9 (6.7, 9.1)	7.0 (6.1, 7.8)	< 0.001	< 0.001
Albumin (g/dL)	3.3 (2.8, 3.7)	3.5 (3.1, 3.9)	3.3 (2.8, 3.7)	3.1 (2.8, 3.3)	< 0.001	< 0.001
Phosphorus (mg/dL)	7.1 (5.9, 8.9)	5.5 (4.8, 6.1)	7.1 (5.9, 8.6)	9.1 (7.4, 10.4)	< 0.001	< 0.001
Serum creatinine (mg/dL)	12.2 (9.9, 15.4)	9.7 (7.9, 11.8)	12.2 (10.0, 15.2)	15.0 (11.8, 19.1)	< 0.001	< 0.001
eGFR (mL/min/1.73m2)	3.4 (2.6, 4.4)	4.2 (3.5, 5.7)	3.4 (2.7, 4.3)	2.7 (2.2, 3.5)	< 0.001	< 0.001
CO_2_ (mmol/L)	16.9 (13.4, 20.6)	22.7 (20.3, 24.7)	17.1 (13.7, 20.2)	14.1 (11.6, 16.5)	< 0.001	< 0.001
Medication profiles, n (%)						
Oral antidiabetic drugs	296 (27.4)	50 (25.5)	182 (27.6)	64 (28.6)	0.771	0.489
Insulin	453 (42.0)	63 (32.1)	264 (40.1)	126 (56.2)	< 0.001	< 0.001
Statin	216 (20.0)	39 (19.9)	135 (20.5)	42 (18.8)	0.854	0.751
Fibrate	31 (2.9)	4 (2.0)	22 (3.3)	5 (2.2)	0.515	0.954
Diuretics	788 (73.0)	128 (65.3)	482 (73.1)	178 (79.5)	0.005	0.001
α-adrenergic agonist	243 (22.5)	32 (16.3)	152 (23.1)	59 (26.3)	0.043	0.016
β-adrenergic antagonists	424 (39.3)	67 (34.2)	270 (41.0)	87 (38.8)	0.230	0.368
Calcium channel blocker	859 (79.6)	128 (65.3)	541 (82.1)	190 (84.8)	< 0.001	< 0.001
ACE inhibitor	331 (30.7)	43 (21.9)	202 (30.7)	86 (38.4)	0.001	< 0.001
Angiotensin receptor blockers	395 (36.6)	69 (35.2)	240 (36.4)	86 (38.4)	0.785	0.494
Antiarrhythmics	65 (6.0)	17 (8.7)	38 (5.8)	10 (4.5)	0.176	0.075
Anticoagulant	37 (3.4)	13 (6.6)	20 (3.0)	4 (1.8)	0.016	0.008
Aspirin	262 (24.3)	54 (27.6)	160 (24.3)	48 (21.4)	0.345	0.145
Antiplatelet	190 (17.6)	38 (19.4)	115 (17.5)	37 (16.5)	0.732	0.447
Acetaminophen	808 (74.9)	135 (68.9)	505 (76.6)	168 (75.0)	0.089	0.175
NSAIDs	183 (17.0)	26 (13.3)	111 (16.8)	46 (20.5)	0.139	0.047
Glucocorticoids	408 (37.8)	70 (35.7)	241 (36.6)	97 (43.3)	0.159	0.098
Beta-lactam antibacterials	565 (52.4)	96 (49.0)	348 (52.8)	121 (54.0)	0.549	0.312
Sulfonamides	27 (2.5)	7 (3.6)	15 (2.3)	5 (2.2)	0.575	0.398
Vancomycin	140 (13.0)	25 (12.8)	83 (12.6)	32 (14.3)	0.805	0.625
Contrast	181 (16.8)	39 (19.9)	105 (15.9)	37 (16.5)	0.424	0.381
Erythropoietin	896 (83.0)	136 (69.4)	566 (85.9)	194 (86.6)	< 0.001	< 0.001
First-year Kt/V_Daugirdas_	1.52 (1.36, 1.74)	1.55 (1.37, 1.80)	1.53 (1.36, 1.74)	1.45 (1.34, 1.65)	0.019	0.007
Types of vascular accesses at dialysis initiation					0.002	NA
Hickman catheterization	794 (88.3)	106 (82.8)	495 (87.3)	193 (94.6)		
Arteriovenous graft/fistula	105 (11.7)	22 (17.2)	72 (12.7)	11 (5.4)		

^a^ p-values are calculated by Kruskal-Wallis test for continuous variables and Chi-square test (or Fisher’s exact test as appropriate) for categorical variables.

^b^ p-values for trend are calculated by Spearman's correlation for continuous variables and by Cochran-Armitage trend test for binary variables.

Abbreviations: eGFR, estimated glomerular filtration rate; CO_2_, carbon dioxide; ACE, angiotensin converting enzyme; NSAIDs, nonsteroidal anti-inflammatory drugs.

**Fig 3:**
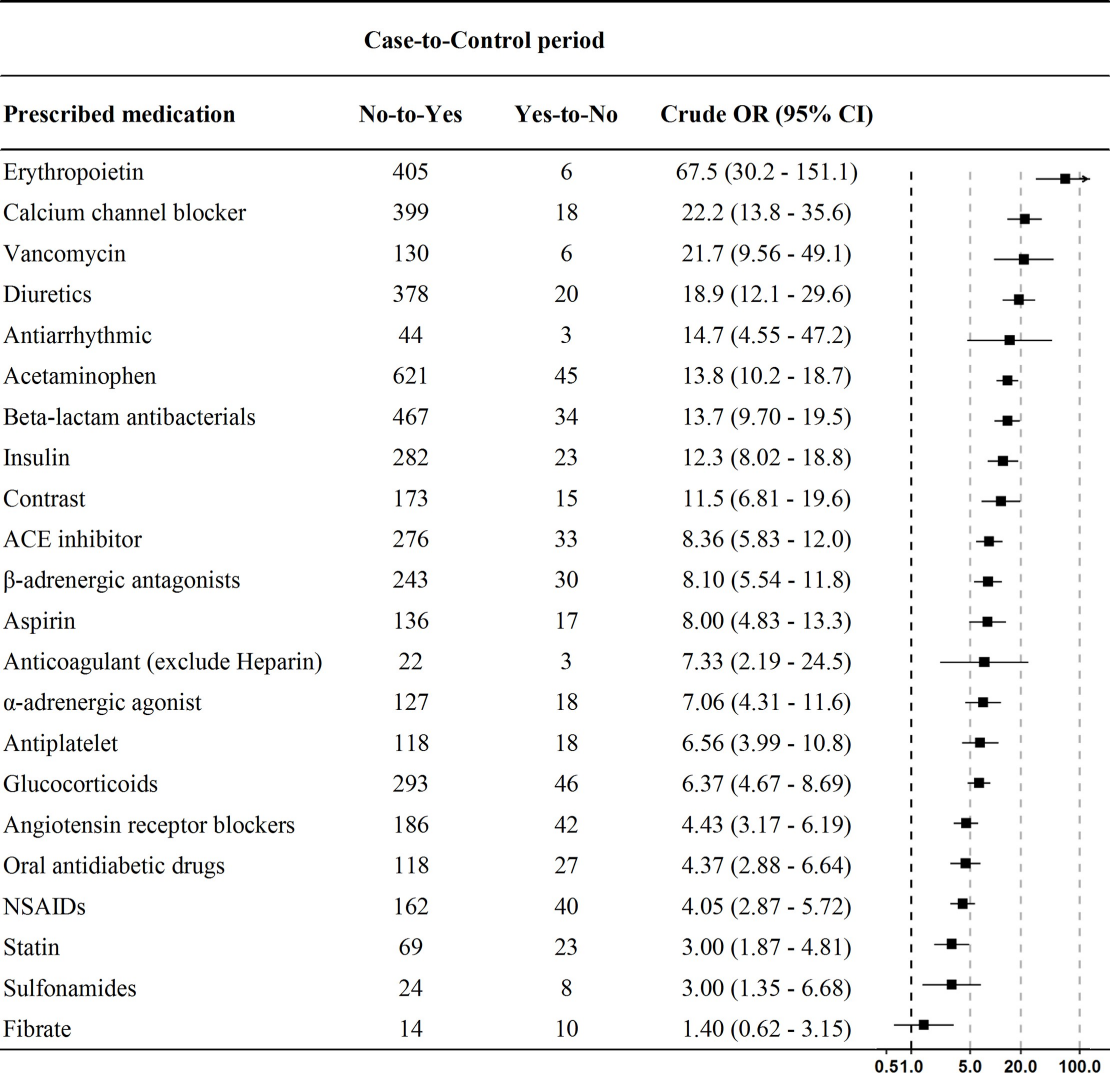
Conditional exposure odds ratio of prescribed medication comparing the utilization pattern between case and control periods. “No” means the medication is not used in the specified period (case or control period), whereas “Yes” means the opposite. The order of these medication follows the exposure odds ratio from high to low.

Compared with patients who were classified in the standard dialysis initiation group, patients in the very late group were much younger and had lower prevalence of CVD but higher prevalence of hypertension (**Table 1**). Other comorbidities such as diabetes and cerebrovascular disease were comparable among the 3 groups of dialysis initiation timing (**Table 1**). Corresponding to what was observed in **Fig 2**, all indicators of dialysis requirement at the time of hemodialysis were worst in the very late group. The very late group was more likely to use insulin, diuretics, angiotensin converting enzyme inhibitors (ACEI), calcium channel blockers (CCBs), anticoagulants, and erythropoiesis-stimulating agents at hemodialysis initiation (**Table 1**). However, the most commonly newly added medication with conditional odds ratios of ≥10.0 for the case periods with a discordant use of medication (newly initiated vs. newly discontinued in the case–control period) were erythropoietin, CCB, vancomycin, diuretics, antiarrhythmic agents, acetaminophen, beta-lactam antibacterials, insulin, and contrast (**Fig 3**). We further analyzed the dose change of furosemide between the case and control periods. The average dose of furosemide used in the case period was 2.5 times higher than that used in the control period. Averagely, patients use 70 mg furosemide daily just before hemodialysis initiation (**S1 Table**).

In multiple Cox proportional hazards regression modeling, the fully adjusted hazards ratios (aHRs) of all-cause mortality for the late and very late groups were 0.97 (95% confidence interval [CI] 0.76–1.23) and 0.89 (0.65–1.21) compared with those in the standard initiation group (**Table 2 Model 3**). In the sensitivity analysis, the observed association remained robust after further adjusting for first-year median Kt/V and the type of vascular accesses on the first dialysis (**Table 2 Model 4**). When we evaluated each indicator separately, persistent anemia (yes-to-yes, 1.68; 95% CI, 1.03–2.75) and hypoalbuminemia (yes-to-yes, 2.00; 95% CI, 1.26–3.18), and newly onset metabolic acidosis (no-to-yes, 1.58; 95% CI, 1.01–2.49) were significantly associated with higher mortality risk after hemodialysis. However, both newly developed and persistent S-Cre of >10 showed protective effects with aHRs 0.59 (95% CI, 0.46–0.76) and 0.58 (95% CI, 0.41–0.82), respectively (**Table 3**).

**Table 2:** Hazard ratios (95% confidence intervals) of all-cause mortality based on the 3 dialysis timing groups.

Dialysis Timing	Case / N	Person-years	Incidence (n per 100 pts/year)	Crude HR (95% CI)	Model 1	Model 2	Model 3	Model 4
Standard	104 / 196	601.34	17.29	Reference	Reference	Reference	Reference	Reference
Late	319 / 659	2054.58	15.52	0.91 (0.73–1.14)	1.03 (0.83–1.30)	1.01 (0.80–1.26)	0.97 (0.76–1.23)	0.97 (0.76–1.24)
Very Late	101 / 224	784.90	12.87	0.75 (0.57–0.99)	0.97 (0.73–1.29)	0.98 (0.74–1.31)	0.89 (0.65–1.21)	0.83 (0.61–1.15)

**Model 1:** Adjusted for age at entry, man, body mass index.

**Model 2:** Further adjusted for diabetes, hypertension, and cardiovascular disease.

**Model 3:** Further adjusted for hemoglobin, serum albumin, and vancomycin.

**Model 4:** Further adjusted for first year median Kt/V and types of vascular accesses.

**Table 3:** Hazard ratios (95% confidence intervals) of all-cause mortality based on the transition patterns of the 7 uremic indicators. “No” means the indicator does not reach the predefined cutoff values, whereas “Yes” means the opposite.

	Case / N	Person-years	Incidence (n per 100 pts/year)	Crude HR (95% CI)	Adjusted HR (95% CI)^a^
**Blood urea nitrogen > 100 mg/dL (*n* = 653)**					
No-to-No	94 / 180	516.76	18.19	Reference	Reference
Yes-to-No	8 / 13	37.41	21.38	1.25 (0.61–2.58)	0.98 (0.45–2.17)
No-to-Yes	163 / 302	909.29	17.93	1.03 (0.80–1.34)	0.81 (0.60–1.11)
Yes-to-Yes	76 / 158	485.81	15.64	0.90 (0.66–1.22)	0.73 (0.51–1.05)
**Potassium > 5.5 mmol/L (*n* = 651)**					
No-to-No	243 / 459	1382.00	17.58	Reference	Reference
Yes-to-No	15 / 36	119.70	12.53	0.70 (0.42–1.19)	0.78 (0.45–1.35)
No-to-Yes	68 / 124	349.65	19.45	1.15 (0.88–1.51)	1.00 (0.73–1.35)
Yes-to-Yes	12 / 32	85.75	13.99	0.81 (0.46–1.45)	0.85 (0.46–1.59)
**Hemoglobin < 9.0 g/dL (*n* = 485)**					
No-to-No	45 / 105	319.57	14.08	Reference	Reference
Yes-to-No	26 / 39	127.04	20.47	1.42 (0.88–2.30)	1.17 (0.68–2.04)
No-to-Yes	78 / 152	491.44	15.87	1.10 (0.76–1.59)	1.15 (0.73–1.83)
Yes-to-Yes	121 / 189	582.29	20.78	1.45 (1.03–2.05)	1.68 (1.03–2.75)
**Albumin < 3.5 g/dL (*n* = 546)**					
No-to-No	70 / 194	493.18	14.19	Reference	Reference
Yes-to-No	22 / 37	96.99	22.68	1.62 (1.00–2.61)	1.63 (0.96–2.75)
No-to-Yes	60 / 113	360.47	16.64	1.10 (0.78–1.56)	1.33 (0.82–2.16)
Yes-to-Yes	129 / 202	636.48	20.27	1.34 (1.00–1.81)	2.00 (1.26–3.18)
**Phosphorus > 6.5 mg/dL (*n* = 572)**					
No-to-No	121 / 227	700.27	17.28	Reference	Reference
Yes-to-No	16 / 33	95.74	16.71	0.99 (0.59–1.67)	0.96 (0.56–1.63)
No-to-Yes	108 / 210	649.14	16.64	1.01 (0.78–1.31)	1.02 (0.74–1.40)
Yes-to-Yes	46 / 102	305.43	15.06	0.90 (0.64–1.26)	1.21 (0.87–1.69)
**Serum creatinine > 10 mg/dL (*n* = 686)**					
No-to-No	130 / 180	465.46	27.93	Reference	Reference
Yes-to-No	3 / 5	15.33	19.57	0.67 (0.21–2.11)	0.94 (0.29–2.99)
No-to-Yes	155 / 332	1045.07	14.83	0.53 (0.42–0.67)	0.59 (0.46–0.76)
Yes-to-Yes	71 / 169	545.02	13.03	0.48 (0.36–0.64)	0.58 (0.41–0.82)
**Bicarbonate < 20 mmol/L (*n* = 368)**					
No-to-No	52 / 116	335.69	15.49	Reference	Reference
Yes-to-No	13 / 24	79.62	16.33	1.11 (0.60–2.06)	0.85 (0.43–1.65)
No-to-Yes	51 / 96	209.86	24.30	1.66 (1.12–2.47)	1.58 (1.01–2.49)
Yes-to-Yes	63 / 132	375.29	16.79	1.13 (0.78–1.64)	0.95 (0.61–1.48)

^a^ Adjusted for age at entry, man, body mass index, diabetes, hypertension, cardiovascular disease, hemoglobin, serum albumin and vancomycin.

## Discussion

This is the first case-crossover study to evaluate prognostic value of dialysis timing determined by the level of uremic burden using objective biochemical indicators. Consistent with prior evidence, among patients with a median eGFR-DI of 3.4 mL/min/1.73 m^2^, we found comparable survival after hemodialysis regardless the timing of dialysis initiation. Main signs/symptoms, speculated by new medication use, that drive the decision of dialysis initiation include difficulty controlling anemia, hypertension, edema, infection, and acute contrast exposure. Among common indicators of dialysis requirement, persistent anemia and hypoalbuminemia are associated with higher all-cause mortality risk.

Our findings support the “safe intent-to-defer” approach even among patients with a very low eGFR, which raises a critical question: can we rely on S-Cre or eGFR to decide the timing of initiation? The answer is clear as we did not find any of these indicators to serve as a single determinant of decompensated uremia. The lack of prognostic roles of biochemical disturbances in mortality is likely confounded by individual’s differential adaptation to chronic uremia. For instance, younger patients may remain free of symptoms such as poor appetite or respiratory distress even with very high S-Cre. By contrast, elder patients may tolerate anemia well due to decreased activity. Indeed, a recent systematic review showed the “safe intent-to-defer” approach conferred a similar survival benefit as dialysis therapy in elder populations [30]. Another unmeasured confounding in this study is the dynamics of these indicators after dialysis. It is possible that patients in the very late group, with at least 6 unfavorable indicators and overall worst values in these indicators, respond better to hemodialysis than those in the standard group. Therefore, dialysis initiation should be based on a shared decision-making process between nephrologists and patients. Mostly, patients make the final call as medication refractoriness is usually defined by patients themselves, and no biomarker can reflect how uremia affects the life quality. Future research efforts should be directed to evaluate the individual's vulnerability to uremic complications and whether these vulnerabilities carry the increased mortality risk into the post-dialysis stage.

In the present study, acute infection is one of the main triggers of dialysis initiation based on the medication use patterns. Acute infection or sepsis can be the last straw that breaks the balance between uremic burden and physiological adaptation. Recent evidence has pointed the mutually aggravated relationship between CKD status and both frequency and severity of acute community-acquired infections [31–33]. Studies have shown that the first 30 days following admission of patients with acute infection are a high-risk period for cardiovascular events, particularly among patients in advanced CKD stages [34, 35]. Infection control and related symptomatic management with antimicrobial agents or analgesics may further induce kidney injury among patients with CKD due to diverse drug-related etiologies such as tubular toxicity, acute interstitial nephritis, and cast nephropathy [36, 37]. More studies are required to evaluate how preventable infections modify the CKD course and how effective implementation of the current infection control policy could translate to ESRD events prevented. Constant vigilance for infection prevention is essential in the multidisciplinary CKD care program.

Diuretics is one of the most frequently observed newly initiated medications right before dialysis initiation in our cohort, usually for hypertension control and volume management. In our study population, the mean dose of loop diuretics was almost doubled during the case period to approximately 70 mg furosemide per day (**S2 Table**). This finding is concordant to a previous study showing diuretics use is associated with an accelerated progression to ESRD [38]. As CKD is a risk multiplier for heart failure from uremia, anemia, and fluid retention, appropriate volume management should be emphasized to avoid cardio-pulmonary-renal decompensation. Further research is required to focus on the predisposing factors that trigger diuretics use in patients with advanced-stage CKD.

It is noteworthy that the odds of NSAIDs use during the case period were over 4 times higher than during the control period. This observation showed that if this avoidable cause of acute kidney injury is eliminated, the dialysis-free period may be extended in approximately 17% of the study population with prevalent NSAIDs exposure in the case period. This prevalence is consistent with a recent study conducted in Poland showing that 16.9% of the study population composed of patients with a wide range of CKDs used NSAIDs [39]. In the United States, a study of a nationally representative sample showed that 10% of the patients with CKD stage 3 and 4 used NSAIDs for more than 30 days prior to study enrollment [40]. They also concluded that the prescription proportion of any and the over-the-counter NSAIDs was not statistically different between patients with (8.1%, any NSAID, and 7.6%, over-the-counter NSAID) and without (8.5% and 8.2%) CKD [41]. Furthermore, another study, based on the Chronic Renal Insufficiency Cohort, found that 24% of the patients with CKD used NSAIDs [42]. Such a prevalent use as this makes developing an effective regulatory strategy to avoid accidental exposure of NSAIDs in patients with CKD, the key priority in CKD care, regardless of whether they are prescribed or over-the-counter purchases.

The present study has several limitations. First, misclassification of the 3 groups with different timings of dialysis initiation could not be completely excluded as we did not have quantifiable information of patient’s subjective symptoms to define major indications of dialysis initiation such as refractory edema or hypertension. Moreover, the cut-off values for the seven proposed laboratory indicators were arbitrary. Robustness and generalizability of these cut-offs remain to be verified. Also, eGFR performance based on the CKD-EPI equation in the Asian population at a very low level may be not accurate [43, 44]. However, other uremic indicators including BUN, phosphate, and serum albumin were consistently getting worse across groups toward very late initiation. Second, physician-level factors such as performance indicators and practice pattern of dialysis initiation were not available. In Taiwan, due to a high incidence and prevalence of ESRD, all nephrologists are required to start dialysis only when patient’s eGFR is <5 ml/min/1.73m^2^ and <10 ml/min/1.73m^2^ in patients with and without diabetes, respectively, along with uremic symptoms. This universal practice pattern prevented our study from providing the whole picture regarding the relation between dialysis timing and survival as our study population only consisted 16 (1.5%) patients with eGFR-DI >10 ml/min/1.73m^2^. Third, conventional imputed approach was not used to correct potential lead-time bias. Instead, our case-crossover design well addressed this issue as the levels of eGFR were comparable among the standard, late, and very late group. Fourth, to minimize survivor bias due to inherent differences of survival rates among standard, late, and very late groups that may lead to overestimation of survival rate, particularly the very late group, we additionally adjusted for health status such as age, comorbidities, and nutritional markers. Finally, the study was conducted in the Han Chinese population, which may limit the generalizability of our findings to other ethnic populations. More research is required to verify whether the ethnicity can influence the associations between dialysis timing and survival outcome.

In conclusion, this is the first study that supports the “safe intent-to-defer” approach among the CKD population with a median eGFR of <5 mL/min/1.73 m^2^. Acute infection prevention through strategized vaccination/immunization planning and optimal fluid management are the keys to safely defer dialysis initiation among patients with a very low eGFR. Moreover, how to minimize accidental NSAID exposure is a critical issue that requires coordinated informatics efforts to develop a real-time alarm system in daily practice. Future randomized experiments are warranted to verify our findings and evaluate the economic impact of the safely deferred approach.

## Supporting information

S1 Table The transition pattern of the 7 common uremic indicators between case and control periods before hemodialysis initiation (N = 1,079). “No” means the indicator does not reach the predefined cutoff values shown on the first column, whereas “Yes” means the opposite. The order of these indicators follows the frequency of “no-to-yes” from high to low.
(DOCX)

S2 Table Distribution of 30-day and daily doses of furosemide in control and case periods and based on the three dialysis timing groups. (DOCX)

S1 Fig Flow diagram of patient selection. CMUH, China Medical University Hospital; HD, hemodialysis.
(DOCX)
