# Supplementary material for: Dialysis timing may be deferred toward very late initiation: An observational study
Source: PLoS One. 2020 May 13;15(5):e0233124. doi: 10.1371/journal.pone.0233124 (PMC7219782; doi:10.1371/journal.pone.0233124)
Supplement: S1 Table — “No” means the indicator does not reach the predefined cutoff values shown on the first column, whereas “Yes” means the opposite. The order of these indicators follows the frequency of “no-to-yes” from high to low. (DOCX) [file pone.0233124.s001.docx]

**Supplementary Table 1.** The transition pattern of the 7 common uremic indicators between case and control periods before hemodialysis initiation (N = 1,079). “No” means the indicator does not reach the predefined cutoff values shown on the first column, whereas “Yes” means the opposite. The order of these indicators follows the frequency of “no-to-yes” from high to low.

|  | **Control-to-Case period** | | | |
| --- | --- | --- | --- | --- |
|  | **No-to-No (%)** | **No-to-Yes (%)** | **Yes-to-Yes (%)** | **Yes-to-No (%)** |
| **Serum creatinine > 10 mg/dL** | 180 (26.2) | 332 (48.4) | 169 (24.6) | 5 (0.7) |
| **Blood urea nitrogen > 100 mg/dL** | 180 (27.6) | 302 (46.2) | 158 (24.2) | 13 (2.0) |
| **Phosphorus > 6.5 mg/dL** | 227 (39.7) | 210 (36.7) | 102 (17.8) | 33 (5.8) |
| **Hemoglobin < 9.0 g/dL** | 105 (21.6) | 152 (31.3) | 189 (39.0) | 39 (8.0) |
| **CO_2_ < 20 mmol/L** | 116 (31.5) | 96 (26.1) | 132 (35.9) | 24 (6.5) |
| **Albumin < 3.5 g/dL** | 194 (35.5) | 113 (20.7) | 202 (37.0) | 37 (6.8) |
| **Potassium > 5.5 mmol/L** | 459 (70.5) | 124 (19.0) | 32 (4.9) | 36 (5.5) |

Abbreviations: CO2, carbon dioxide.
