# Supplementary material for: Dialysis timing may be deferred toward very late initiation: An observational study
Source: PLoS One. 2020 May 13;15(5):e0233124. doi: 10.1371/journal.pone.0233124 (PMC7219782; doi:10.1371/journal.pone.0233124)
Supplement: S2 Table — (DOCX) [file pone.0233124.s002.docx]

**Supplementary Table 2.** Distribution of 30-day and daily doses of furosemide in control and case periods and based on the three dialysis timing groups.

| **Furosemide doses** (mg) | **Control period** | | | |  | **Case period** | | | |
| --- | --- | --- | --- | --- | --- | --- | --- | --- | --- |
|  | **Total** | **Standard** | **Late** | **Very Late** |  | **Total** | **Standard** | **Late** | **Very Late** |
| 30-day |  |  |  |  |  |  |  |  |  |
| Mean ± SD | 809 ± 1734 | 715 ± 1316 | 886 ± 1945 | 662 ± 1346 |  | 2096 ± 2855 | 1861 ± 2995 | 2097 ± 2899 | 2299 ± 2580 |
| Median (IQR) | 0 (0 - 1160) | 0 (0 - 1140) | 0 (0 - 1200) | 0 (0 - 600) |  | 960 (0 - 2987) | 544 (0 - 2500) | 800 (0 - 2960) | 1360 (160 - 3666) |
| Daily |  |  |  |  |  |  |  |  |  |
| Mean ± SD | 27 ± 58 | 24 ± 44 | 30 ± 65 | 22 ± 45 |  | 70 ± 95 | 62 ± 100 | 70 ± 97 | 77 ± 86 |
| Median (IQR) | 0 (0 - 39) | 0 (0 - 38) | 0 (0 - 40) | 0 (0 - 20) |  | 32 (0 - 100) | 18 (0 - 83) | 27 (0 - 99) | 45 (5 - 122) |
