# Supplementary material for: Dialysis timing may be deferred toward very late initiation: An observational study
Source: PLoS One. 2020 May 13;15(5):e0233124. doi: 10.1371/journal.pone.0233124 (PMC7219782; doi:10.1371/journal.pone.0233124)
Supplement: S1 Fig — CMUH, China Medical University Hospital; HD, hemodialysis. (DOCX) [file pone.0233124.s003.docx]

**Supplementary Figure 1.** Flow diagram of patient selection. CMUH, China Medical University Hospital; HD, hemodialysis

1,079 subjects were included for statistical analysis

1,718 subjects covered by HD database

in CMUH (2006-2015)

639 subjects being excluded

- Without identification number (n = 1)
- Initial dialysis is not at CMUH (n = 139)
- Initial dialysis date is before entry date (n = 484)
- Age greater than 90 or less than 18 (n = 13)
- Missing records of initial dialysis date (n = 2)
